# The development and validation of a nomogram to determine neurological outcomes in cardiac arrest patients

**DOI:** 10.1186/s12871-023-02251-5

**Published:** 2023-08-24

**Authors:** Xuru Zhang, Xiaowei Zheng, Zhisen Dai, Huizhe Zheng

**Affiliations:** 1 Department of Anesthesiology, Clinical Oncology School of Fujian Medical University, Fujian Cancer Hospital, Fuzhou City, 350014 China No 420 Fuma Road, Jinan District, Fujian Province; 2 Department of Otorhinolaryngology Head and Neck Surgery, Shengli Clinical Medical College of Fujian Medical University, Fuzhou, 350001 China

**Keywords:** Neurological outcome, Cardiac arrest, Nomogram, Previous neurological disease, CPR

## Abstract

**Objectives:**

This study aimed to investigate the variables that influence neurological functional restoration in cardiac arrest patients and construct a nomogram to predict neurofunctional prognosis.

**Patients and methods:**

We extracted the data from the Dryad database. Associations between patient variables and neurological outcomes were examined by logistic regression models. On the basis of these predictors, a prognostic nomogram was constructed. The identification and calibration of the prognostic nomogram were evaluated through the receiver operating characteristic (ROC) curve, the calibration curve, and the concordance index (C-index).

**Results:**

A total of 374 cardiac arrest individuals were recruited in the research. Sixty percent of the participants had an adverse neurological result. The multivariable logistic regression analysis for poor neurological recovery, which showed patient age ≥ 65 years, previous neurological disease, witnessed arrest, bystander cardio-pulmonary resuscitation(CPR), cardiac arrest presenting with a non-shockable rhythm, total epinephrine dose ≥ 2.5 mg at the time of resuscitation and acute kidney injury(AKI) remained independent predictors for neurological outcomes.

**Conclusions:**

The novel nomogram based on clinical characteristics is an efficient tool to predict neurological outcomes in cardiac arrest patients, which may help clinicians identifying high-risk patients and tailoring personalized treatment regimens.

## Introduction

Cardiac arrest is one of the leading causes of death globally, with a universal prevalence of 5 to 11 cases per 100,000 inhabitants annually [1, 2]. It has long been believed that the prognosis of cardiac arrest were so poor that resuscitation may not even be necessary. While results remain disappointing, more recent findings indicate that progress has been made over the last two decades [3].

Neurological dysfunction, which primarily stems from global ischemia–reperfusion injury, is the determining factor that contributes to adverse outcomes following cardiac arrest [4]. Although targeted temperature management (TTM) and other neuroprotective strategies have made significant strides, the outcomes of discharge from the hospital remain unsatisfactory, and the favorable neurological recovery rate is only about 8% [5]. More recently, the prediction of neurological function in comatose individuals following cardiopulmonary resuscitation(CPR) is the hot spot of research. Several prognostic methods, including electroencephalography, visual and quantitative brain magnetic resonance imaging, somatosensory evoked potential, neuron-specific enolase, serum tau, grey matter to white matter ratio in brain, and optic nerve sheath diameter, have been applied to predicting the neurological outcomes for individuals who suffered cardiac arrest [6–11]. Nonetheless, these techniques are not consistently accessible, and predicting neurological functional restoration conditions for post-cardiac arrest individuals remains challenging.

Researchers consider that nomogram, which is a simple graphical representation of statistical prediction model, to be an effective and practical method for recently predicting the survival and prognosis of individuals experiencing cardiac arrest [12, 13]. Nevertheless, to the best of our knowledge, no prognostic nomograms for recovery of neurological function in comatose individuals following cardiac arrest have been established. Using the Dryad database, the present study was the first to establish a comprehensive prognostic nomogram to predict neurological outcomes for comatose individuals following cardiac arrest.

## Methods

It is a secondary analysis of the clinical data from a retrospective trial conducted by Iesu et al. The complete study data can be downloaded through the Dryad database (via: https://doi.org/10.5061/dryad.qv6fp83). The Dryad database is a repository of quality data resources that make the data behind scientific publications discoverable, reusable and citable. Using the Dryad database, researchers can access data, study and validate published data, or solve new problems. In particular, the Dryad database is free of charge without registration. The database allows users to freely download raw data. According to the Dryad Terms of Service, the paper's accessible data can be used to re-evaluate various scientific hypotheses [14, 15]. The previous investigation was initially carried out in the Erasme Hospital, Brussels, Belgium, between January 2007 to December 2015, and the study protocol was approved by the Comite' d'Ethique Hospitalo-Facultaire Erasme-ULB (P2017/264), in accordance with the Declaration of Helsinki.

### Patient enrollment

#### Inclusion and exclusion criteria

Patients who had been hospitalized after an out-of-hospital cardiac arrest (OHCA) or experienced an in-hospital cardiac arrest (IHCA) with a Glasgow Coma Scale (GCS) of less than nine were enrolled in the initial investigation. Exclusion criteria were as follows: liver function results were missing, or death less than 24 h after ICU admission According to a standard institutional post-resuscitation care treatment that has been thoroughly detailed elsewhere, all participants were routinely administered a targeted temperature control of 32 to 34 °C for 24 h.

### Information collection

In the prior investigation, Iesu et al. assembled baseline demographic information, comorbidities (such as hypertension, diabetes, chronic obstructive pulmonary disease, asthma, chronic heart failure, chronic renal failure, liver cirrhosis, and previous neurological disease), as well as clinical characteristics at the time of CPR (including witnessed arrest, bystander CPR, time to restoration of spontaneous circulation, non-electrically shockable rhythm, cardiac arrest of non-cardiac origin, being in shock during ICU, vasopressor drugs therapy, and total epinephrine dose) in all patients. The definition of neurological disease in our study, according to the National Institute of Neurological Disorders and Stroke https://www.ninds.nih.gov/disorders/all-disorders.

### Follow-up and neurological prognosis

The main outcome was the quality of neurological functional restoration, which was evaluated three months after cardiac arrest using the cerebral performance category (CPC) score. CPC, a well-validated prognostic tool frequently used in clinical settings [16, 17]. CPC levels 1 (no or mild neurological disability) and 2 (moderate neurological impairment) were thought to indicate a favorable neurological prognosis, whilst CPC levels 3 (serious neurological impairment), 4 (persistent vegetative state), and 5 (death) were classified as a poor prognosis. Prospectively, the CPC was carried out at follow-up visits or over the phone with the general practitioner.

### Statistical analysis

For the statistical analysis, SPSS software (IBM SPSS Statistics Version 25.0) and R 4.1.3 software (RStudio Version 1.3.1093) were employed. The initial determination of the normality of the distribution of continuous data was made using the Shapiro–Wilk and Kolmogorov–Smirnov tests. While parametric data were reported as mean with standard deviation and compared between groups using the Independent-samples t-test, nonparametric variables were supplied as median and compared between groups using the Mann–Whitney U test. When appropriate, categorical variables were assessed using Fisher's exact or the χ^2^ test and given as numbers (percentage). By using univariate and multivariable models, logistic regression analysis was used to evaluate the relationship between clinical variables and neurological outcomes. For each component that was included, a univariate analysis was conducted, and all indicators from this analysis were then included in a multivariate logistic regression analysis using the forward stepwise logistic regression with a 0.05 inclusion and exclusion threshold. 95% confidence intervals (CI) for the odds ratio (OR) were employed for the logistic regression models.

The “rms” and “DynNom” packages were utilized to construct a static and dynamic nomogram, respectively. The “rms” is a collection of functions that assist with and streamline modeling (https://CRAN.R-project.org/package=rms). It also contains functions for binary and ordinal logistic regression models, ordinal models for continuous Y with a variety of distribution families, and the Buckley-James multiple regression model for right-censored responses, and implements penalized maximum likelihood estimation for logistic and ordinary linear models. 'rms' works with almost any regression model, but it was especially written to work with binary or ordinal regression models, Cox regression, accelerated failure time models, ordinary linear models, the Buckley-James model, generalized least squares for serially or spatially correlated observations, generalized linear models, and quantile regression. During the internal validation of the nomogram, a concordance index (C-index) was generated by assessing the area under the curve (AUC) of the receiver operating characteristic (ROC) curve. In existing studies, the C-index is the most common and widely recognized indicators for evaluating the predictive performance of models [18, 19]. C-index can be used to judge the accuracy and discrimination ability for global prediction, and the performance is relatively stable. Therefore, we chose C-index to evaluate the predictive performance of the nomogram. The C-index is between 0.5 and 1 (the probability of agreement and disagreement is exactly 0.5 for any random pair). 0.5 means that the model has no predictive effect, while 1 means that the model is in complete agreement. In general, a C-index between 0.50 and 0.70 is considered to be of low accuracy, between 0.71 and 0.90 is considered to be of medium accuracy, and above 0.90 is considered to be of high accuracy [20]. All reported *P* values are two-tailed, and a *P* value of less than 0.05 was considered statistically significant.

## Results

### Patient characteristics

A total of 435 IHCA and OHCA individuals were recruited in the previous research, including 125(28.70%) OHCA and 310(71.30%) IHCA, of which sixty-one patients were removed as early Mortality (*n* = 51) or coagulation, liver transaminases, or total bilirubin was not documented at the time of admission (*n* = 10). The final analysis comprised 374 patients with IHCA and OHCA. Three months after cardiac arrest, approximately sixty percent of the study participants had an adverse neurological result. We have concluded the baseline demographics, clinical characteristics, and prognosis in Table 1.

**Table 1:** Clinical characteristics of patients with cardiac arrest

Variables	Total	Favorable neurological outcome(*N* = 148)	Poor neurological outcome(*N* = 226)	*P*
Male	270(72.19)	109(73.65)	161(71.24)	0.611
Age, years	62(51–74)	58(50.25–70.75)	66(52–76)	0.002
Weight, kg	77(67–85)	78(70–85.75)	76(65–85)	0.164
**Comorbidity**				
Hypertension	159(42.51)	65(43.92)	94(41.59)	0.656
Diabetes	91(24.33)	30(20.27)	61(26.99)	0.139
Coronary artery disease	146(39.04)	55(37.16)	91(40.27)	0.547
COPD/Asthma	63(16.84)	19(12.84)	44(19.47)	0.094
Chronic Heart Failure	78(20.86)	28(18.92)	50(22.12)	0.456
Chronic Renal Failure	62(16.58)	21(14.19)	41(18.14)	0.315
Liver cirrhosis	17(4.55)	3(2.03)	14(6.19)	0.058
Previous Neurol Disease	54(14.44)	13(8.78)	41(18.14)	0.012
**Clinical characteristics at the time of CPR**				
Witnessed arrest	320(85.56)	136(91.89)	184(81.42)	0.005
Bystander CPR	254(67.91)	114(77.03)	140(61.95)	0.002
Time to ROSC, min	15(7–25)	11.5(5–20)	17(10–25)	< 0.001
Non-cardiac etiology	153(40.91)	49(33.11)	104(46.02)	0.014
None-shockable rhythm	221(59.09)	61(41.22)	160(70.80)	< 0.001
**During ICU stay**				
Shock	200(53.48)	64(43.24)	136(60.18)	0.001
Vasopressor therapy	283(75.67)	100(67.57)	183(80.97)	0.003
AKI	221(59.10)	70(47.30)	151(66.81)	< 0.001
Mechanical ventilation	369(98.66)	143(96.62)	226(100)	0.005
IABP	24(6.42)	6(4.05)	18(7.96)	0.131
ECMO	47(12.57)	18(12.16)	29(12.83)	0.848
CRRT	61(16.31)	21(14.19)	40(17.70)	0.369
**Outcome**				
ICU stay, day	4(2–9)	6(3–11.75)	3(2–8)	< 0.001
ICU death	194(51.87)	0(0)	194(85.84)	< 0.001
In-hospital death	213(56.95)	0(0)	213(94.25)	< 0.001

Abbreviations: *COPD* Chronic obstructive pulmonary disease, *CPR* Cardiopulmonary resuscitation, *ROSC* Restoration of spontaneous circulation, *ICU* Intensive care unit, *AKI* Acute Kidney Injury, *IABP* Intra-aortic ballon pump, *ECMO* Extracorporeal membrane oxygenation, *CRRT* Continuous renal replacement therapy

### Univariate and multivariate regression analysis

According to the univariate logistic regression model, patient age, previous neurological disease, previous history of corticosteroid use, witnessed arrest, bystander CPR, ROSC time, cardiac arrest of non-cardiac origin, cardiac arrest presenting with a non-shockable rhythm, blood lactate on admission, total epinephrine dose at the time of resuscitation, and being in shock during ICU, acute kidney injury(AKI), and receipt of vasopressor drugs were all significantly associated with adverse neurological outcomes. All of the variables were then incorporated into a multivariable logistic regression analysis for poor neurological recovery, which showed patient age ≥ 65 years (OR = 2.614, 95%CI = 1.501–4.552, *P* = 0.001), previous neurological disease (OR = 3.002, 95%CI = 1.348–6.687, *P* = 0.007), witnessed arrest (OR = 0.391, 95%CI = 0.172–0.887, *P* = 0.025), bystander CPR (OR = 0.502, 95%CI = 0.279–0.903, *P* = 0.021), cardiac arrest presenting with a non-shockable rhythm (OR = 2.706, 95%CI = 1.549–4.729,* P* < 0.001), total epinephrine dose ≥ 2.5 mg at the time of resuscitation (OR = 2.018, 95%CI = 1.072–3.800,* P* = 0.030) and AKI (OR = 2.043, 95%CI = 1.171–3.565,* P* = 0.012)remained independent predictors for poor neurological outcomes. Univariate and multivariate logistic regression analyses of predictors of adverse neurological outcomes are shown in Table 2.

**Table 2:** Univariate and multivariate analysis for neurological outcome three months after cardiac arrest

Variable	Univariate analysis			Multivariate analysis		
	**OR**	**95%CI**	***P***	**OR**	**95%CI**	***P***
**Gender**	0.886	0.556–1.412	0.611	/	/	/
**Age ≥ 65 year**	2.029	1.326–3.106	0.001	2.614	1.501–4.552	0.001
**Hypertension**	0.909	0.598–1.382	0.656	/	/	/
**Diabetes**	1.454	0.885–2.390	0.140	/	/	/
**COPD/Asthma**	1.641	0.916–2.942	0.096	/	/	/
**Chronic Heart Failure**	1.218	0.726–2.043	0.456	/	/	/
**Chronic Renal Failure**	1.340	0.756–2.376	0.316	/	/	/
**Liver cirrhosis**	3.192	0.901–11.306	0.072	/	/	/
**Previous Neurol Disease**	2.301	1.187–4.462	0.014	3.002	1.348–6.687	0.007
**Corticoids**	1.778	1.056–2.996	0.031	/	/	/
**Chronic Anticoagulation**	0.979	0.567–1.690	0.938	/	/	/
**Witnessed arrest**	0.387	0.196–0.762	0.006	0.391	0.172–0.887	0.025
**Bystander CPR**	0.486	0.3040.0775	0.002	0.502	0.279–0.903	0.021
**Time to ROSC ≥ 17 min**	2.257	1.461–3.487	< 0.001	/	/	/
**Non-cardiac cause**	1.722	1.119–2.650	0.013	/	/	/
**None-shockable rhythm**	3.458	2.238–5.342	< 0.001	2.706	1.549–4.729	< 0.001
**Shock**	1.983	1.303–3.020	0.001	/	/	/
**Vasopressor therapy**	2.043	1.266–3.296	0.003	/	/	/
**AKI**	2.243	1.466–3.432	< 0.001	2.043	1.171–3.565	0.012
**Lac ≥ 6 mmol/L**	1.893	1.211–2.958	0.005	/	/	/
**Epinephrine dose ≥ 2.5 mg**	2.516	1.641–3.859	< 0.001	2.018	1.072–3.800	0.030

Abbreviations: *COPD* Chronic obstructive pulmonary disease, *CPR* Cardiopulmonary resuscitation, *ROSC* Restoration of spontaneous circulation, *AKI* Acute Kidney Injury, *OR* Odds ratio, *95%CI* 95% Confidence interval; / means no data

### Construction of the nomogram

**Fig. 1:**
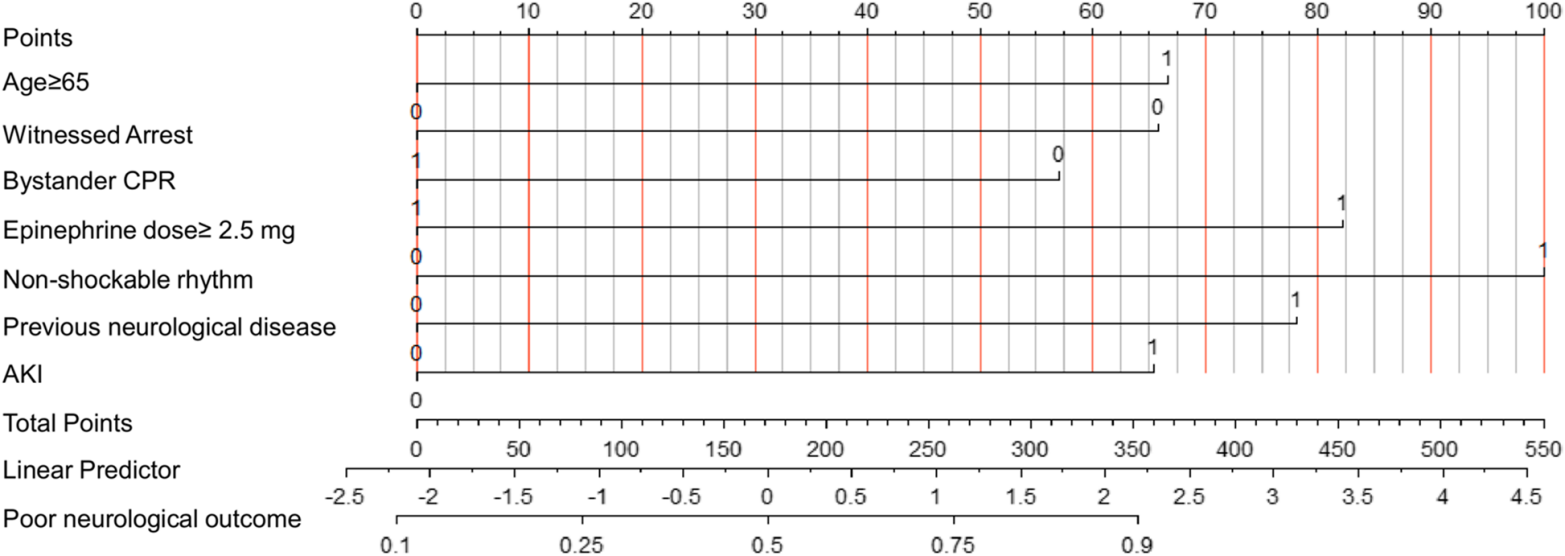
A constructed nomogram for neurological prognostic prediction of a patient with cardiac arrest. The nomogram is used by summing all points identified on the scale for each variable. The total points projected on the bottom scales indicate the probabilities of poor neurological outcomes three months after cardiac arrest

Based on those mentioned above seven independent indicators, this study constructs a nomogram of neurological prognosis in patients who have experienced cardiac arrest (Fig. 1). Concurrently, we established a risk assessment online tool, which is available through the URL: https://poorneurologicaloutcome.shinyapps.io/neurological_outcomes/. We can individually predict the probability of poor neurological recovery after three months for each patient in a cardiac arrest coma, for instance: For example, a patient over 65 years of age who had a witnessed cardiac arrest and was resuscitated by a bystander, resuscitated with a total epinephrine dose greater than 2.5 mg, was in a non-electric shockable rhythm, had no previous neurological disease, and did not have AKI during ICU, had a 71.9% probability of an adverse neurological prognosis.

### Validation of the nomogram

**Fig. 2:**
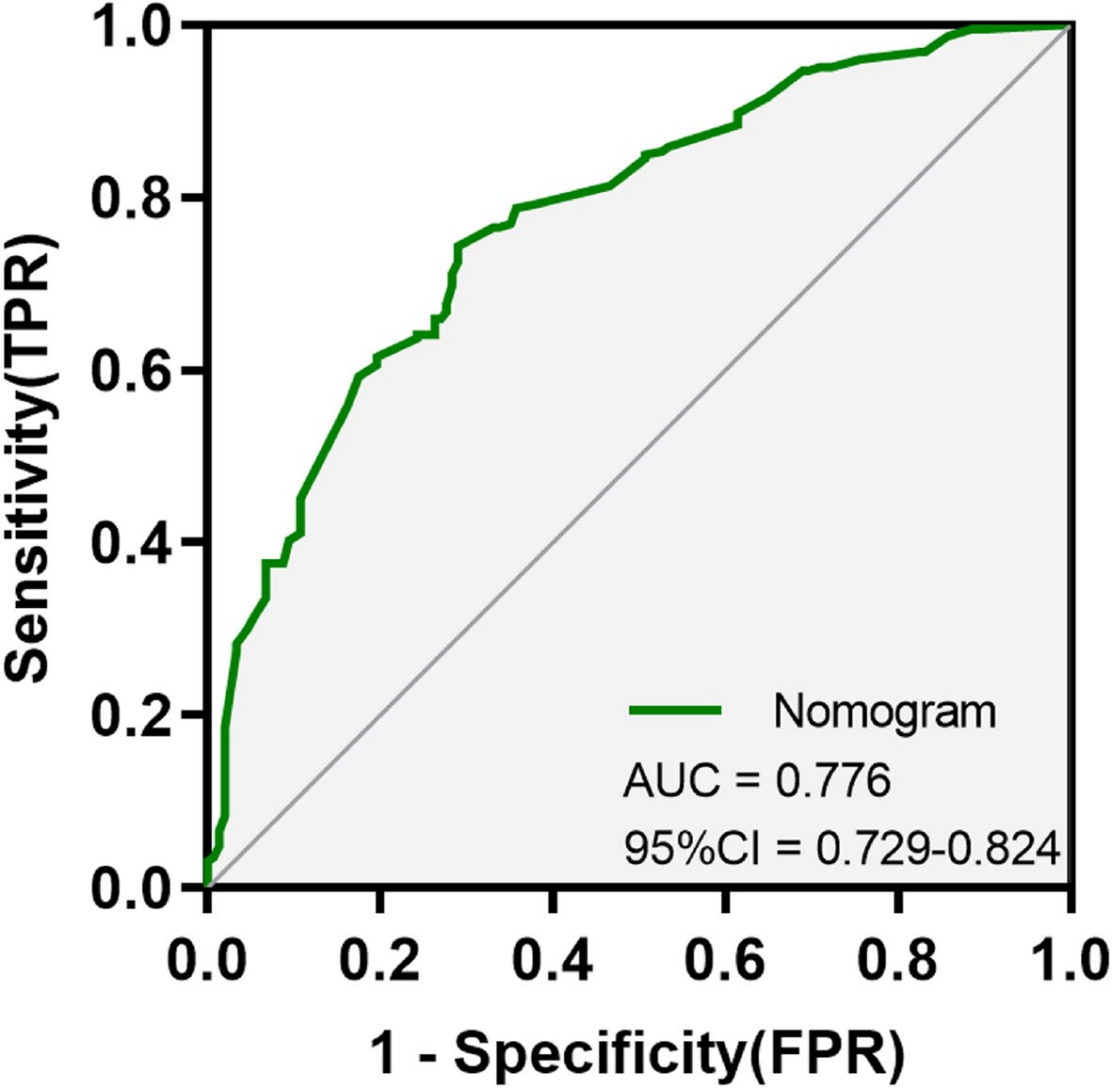
ROC curves to verify accurate predictability for poor neurological outcomes three months after cardiac arrest

**Fig. 3:**
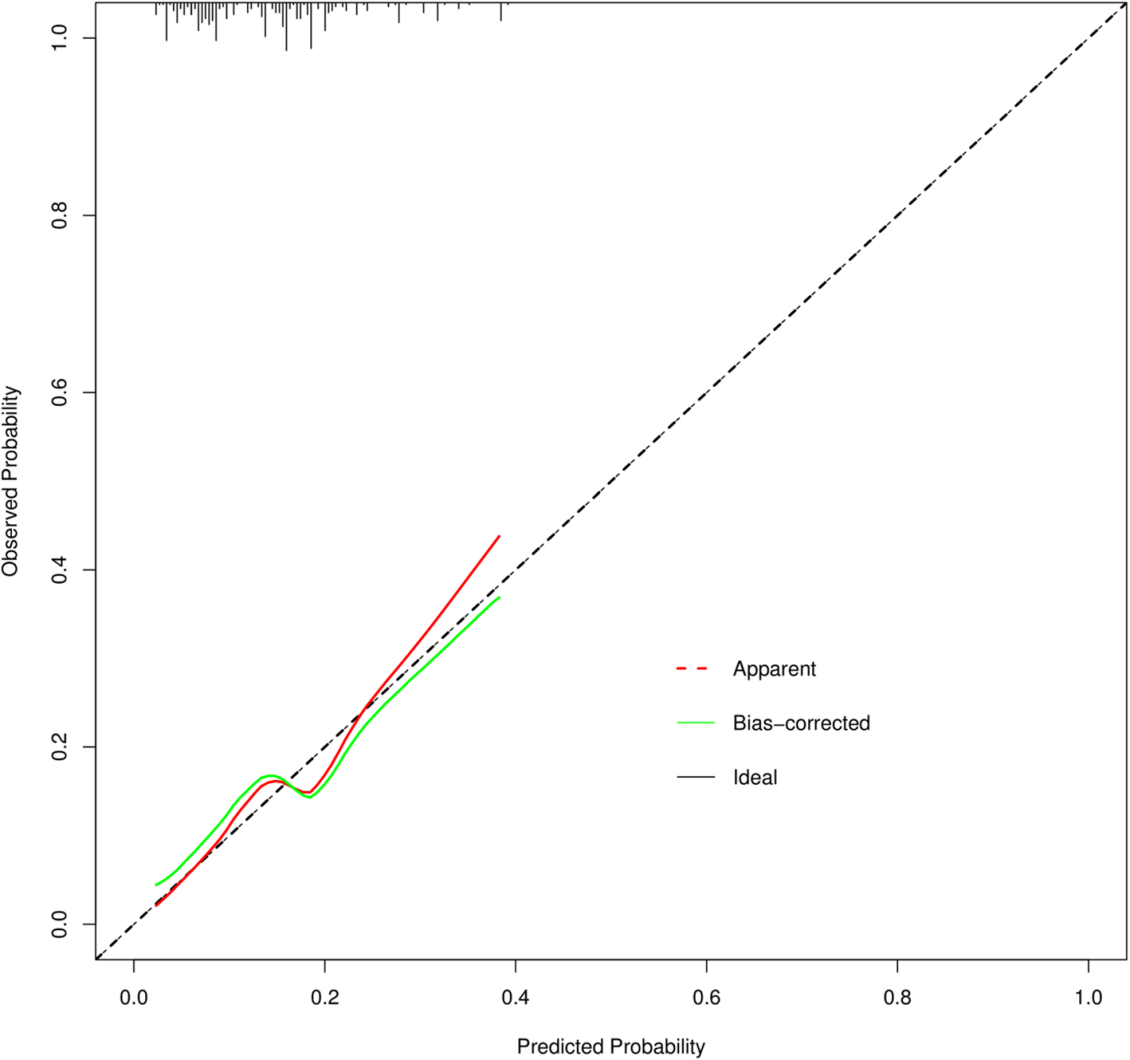
The calibration curve for predicting neurological outcome three months after cardiac arrest The diagonal dashed line shows the exact match between the anticipated values of the nomogram and the observed probabilities. The values on the X-axis reflect the predicted values of the nomogram, and the values on the Y-axis indicate the actual probabilities

In this study, the accuracy of the prediction nomogram was generated using the ROC curve, Hosmer–Lemeshow goodness-of-fit test, the calibration curve, and the C-index. The AUC was utilized to assess the prediction capacity for discrimination. AUC was 0.776 (95% CI: 0.729–0.824), with a sensitivity of 74.30% and a specificity of 70.90%, and the positive predictive value and negative predictive value were 79.60% and 64.40%, respectively (Fig. 2). The results demonstrated that the nomogram prediction model had a remarkable discriminatory accuracy. According to the results of the Hosmer–Lemeshow goodness-of-fit test, the difference between the predicted risk and the actual probability of occurrence was not statistically significant (Chi-square = 0.818, *P* = 0.664). Additionally, the Bootstrap internal validation method was used to validate the Nomogram scoring system with a self-sampling of 1000 times, and the calibration curve was plotted. The nomogram showed excellent discrimination accuracy for the prediction of a poor neurological prognosis in the internal validation, and the C-index value was 0.679 (95% CI: 0.604–0.754) (Fig. 3).

## Discussion

The object of this investigation was to probe independent factors from patient clinical variables and resuscitation characteristics that may be utilized for early risk stratification and prediction of adverse neurological results in cardiac arrest survivors who were unconscious. In the present investigation, we demonstrated that seven risk variables were significant predictors of neurological outcomes, created a simple nomogram to effectively predict neurological prognosis, and verified the predictability of the model internally. The prognostic nomogram can be used to assess the neurological outcomes of each cardiac arrest individual, and may enable clinicians to consider personalized treatment regimens for patients at high-risk.

Of the patient-related variables, advanced age and a history of neurological disease were both prominently correlated with neurological outcomes in a multivariable model. Our findings are aligned with prior investigations confirming an adverse neurological outcome in geriatric patients who suffered cardiac arrest [21, 22]. The prevailing consensus is that geriatric patients, especially those with numerous comorbidities, may experience poor neurologic outcomes following CPR for cardiac arrest. This is confirmed by the results of former researchers such as Sender et al., who revealed that elderly patients over seventy-five years of age were more likely to have poor neurological functional results six months after discharge upon reviewing the information from cardiac arrest individuals who survived in six interventional cardiology centers over the course of a five-year research period [23]. Ester et al. retrospectively analyzed 1,285 adult cardiac arrest participants on the department of ICU from 2005 to 2013, then indicated that elderly ICU-treated cardiac arrest patients had worse neurofunctional restoration and a higher proportion of deaths than younger patients after [24]. Moreover, we have found that a history of a previous neurological disease was a powerful prognostic predictor associated with adverse outcomes. Patients with previous neurological disease produced a greater likelihood of achieving poor neurological prognosis compared with those without (OR = 3.002, 95%CI = 1.348–6.687, *P* = 0.007). We hypothesize that the pre-existence of neurological disease may exacerbate the global-brain ischemia–reperfusion injury that results from cardiac arrest followed by CPR and subsequent restoration of natural circulation.

Consistent with existing literature, we determined that resuscitation features, particularly the existence of an initial shockable rhythm, witnessed arrest, and bystander CPR were strong predictors of prognosis [25, 26]. In contrast to patients who presented with non-shockable rhythm, previous studies demonstrated that neurocognitive prognoses following cardiac arrest were far more favorable in patients with initial shockable rhythm [27]. This would imply that patients with non-shockable rhythms were assumed to have un-reversible causes of heart disease apart from myocardial ischemia [28].

In line with others, the findings of the multivariate logistic regression model analysis in our research revealed that the occurrence of AKI was an independent risk indicator for poor neurological prognosis in post-cardiac arrest patients. The risk of causing a poor neurological outcome after cardiac arrest was two-fold higher in patients with AKI than in non-AKI. C Storm et al. proved that in a ten-year observational follow-up of 503 patients with cardiac arrest who received TTM control, the occurrence of AKI during ICU stay was a considerable risk factor for impaired neurological function and greater mortality following cardiac arrest [29]. This was validated in a study by Oh et al., where AKI was linked to poor neurological recovery at six months in OHCA patients receiving TTM [30].

It is confirmed in the 2015 ILCOR guidelines for advanced cardiovascular life support that intravenous adrenaline is a core pharmacologic intervention during resuscitation from cardiac arrest [31]. Our study detected that individuals who accepted a total dosage of more epinephrine than 2.5 mg were more likely to have an adverse neurological prognosis. These findings are in concordance with a prospective, randomized, observational study among 417,188 OHCAs occurring in Japan. In that study, Hagihara et al. found that the epinephrine-treated group had a significantly lower likelihood of 1-month survival and positive neurological recovery [32]. One plausible explanation for this condition is that epinephrine improves the success of resuscitation by stimulating α- and β-adrenergic receptors, thus increasing cardiac perfusion during cardiopulmonary resuscitation. In order to provide this transitory benefit for coronary infusion, blood stream to all other organs, as well as the brain, is simultaneously decreased. Despite the improvement in ROSC, this impact may persist even after pulse recovery and eventually lead to the accumulation of metabolic debt in the body and brain, which adversely affects long-term outcomes [31].

In the present investigation, we structured a nomogram to predict the neurological functional restoration of individuals with cardiac arrest based on several clinical parameters. In comparison with single risk indicators, the prediction model on the basis of multiple risk indicators is further capable of aiding the clinician in noticing the patients who are vulnerable to adverse neurological outcomes. Seven prognostic indicators are strongly linked with neurological prognosis, according to the multivariate logistic regression analysis. With an internally validated C-index of 0.679 (95% CI: 0.604–0.754) and the mean error of the internal validation test was less than 2%, the nomogram specifically showed excellent discrimination accuracy, indicating that the model has an effective predictive performance.

A possible limitation of our study is that bias might arise due to the fact that neurological recovery conditions were only assessed three months after cardiac arrest and failed to conduct long-term follow-up. Second, further study was needed to back up our conclusions as our model could only be internally verified, and the nomogram has not been verified in the external validation cohort. Our research team is collecting patients with in-hospital cardiac arrest who are treated at our center after 2023. An external validation cohort made from patients who experience in-hospital cardiac arrest at our center after 2023 would not be ideal because i) our center is a specialist oncology hospital, and most of the patients who experience in-hospital cardiac arrest are patients with advanced malignancies, therefore, the source of patients is relatively limited, ii) it would introduce a bias due to the lack of emergency departments for out-of-hospital cardiac arrests in our center. The estimation of the clinical importance of our theory will require validation in a multicenter external cohort. We have taken preliminary contacts with other comprehensive tertiary centers, and future studies engaging larger participant cohorts will be required to testify these findings.Third, we analyzed a mixed population of patients with OHCA and IHCA, who may have different predisposing conditions for the development of adverse neurological outcomes. However, this is also a limitation of the study, because the sample size was insufficient to allow separate analysis of the cohorts. Our study population was predominately IHCA patients, therefore, the nomogram may be more reasonably reflective of IHCA rather than OHCA. Fourth, the withdrawal of life support in patients who were treated in ICU after cardiac arrest may have an impact on the nomogram in terms of producing a self-fulfilling prophecy. The percentage of withdrawal of life support is a major issue in this study that unfortunately could not available for analysis since this information was not collected. Future research should focus on this important issue.

## Conclusions

Utilizing seven independent predictive features, we have constructed a prognostic nomogram that successfully predicts neurofunctional recovery three months after cardiac arrest in comatose individuals. Nomograms may be instrumental in risk discrimination and personal clinically-based interventions.

## Data Availability

The datasets generated and/or analysed during the current study are available in the Dryad database repository, https://doi.org/10.5061/dryad.qv6fp83.
